# Quantitative splenic embolization possible: application of 8Spheres conformal microspheres in partial splenic embolization (PSE)

**DOI:** 10.1186/s12876-021-01991-3

**Published:** 2021-10-27

**Authors:** Haohao Lu, Chuansheng Zheng, Bin Liang, Bin Xiong

**Affiliations:** 1 Department of Radiology, Wuhan Union Hospital, Tongji Medical College, Huazhong University of Science and Technology, Jiefang Avenue #1277, Wuhan, 430022 China; 2 Hubei Province Key Laboratory of Molecular Imaging, Wuhan, 430022 China

**Keywords:** 8Spheres, Partial splenic embolization, PSE, Hypersplenism, Microspheres, Adverse events, Thrombocytopenia, Leukopenia

## Abstract

**Background:**

To investigate the safety and efficacy of 8Spheres in partial splenic embolization. To explore the possibility of accurate control of splenic embolic volume by quantifying the number of microspheres used during PSE.

**Method:**

The data of 179 patients who underwent PSE were collected. The patients were divided into two groups: 300–500 um microsphere group (N = 83) and 500–700 um microsphere group (N = 96). The spleen volume before PSE, infarct volume and infarct rate of the spleen after PSE, changes in peripheral blood cells after PSE, postoperative adverse events and incidence of infection were compared between the two groups.

**Results:**

300–500 um group vs 500–700 um group: postoperative spleen volume (cm^3^): 753.82 ± 325.41 vs 568.65 ± 298.16 (P = 0.008); spleen embolization volume (cm^3^): 525.93 ± 118.29 vs 630.26 ± 109.71 (P = 0.014); spleen embolization rate: 41.1 ± 12.3% vs 52.4 ± 10.1% (P = 0.021). Leukocytes and platelets were significantly increased after PSE in both groups; leukocyte, 1 month: 4.13 ± 0.91 vs 5.08 ± 1.16 (P = 0.026); 3 months: 4.08 ± 1.25 vs 4.83 ± 0.98 (P = 0.022); platelet, 1 month: 125.6 ± 20.3 vs 138.7 ± 18.4 (P = 0.019); 3 months: 121.8 ± 16.9 vs 134.3 ± 20.1 (P = 0.017). Incidence of abdominal pain after PSE, 72 (86.7%) vs 69 (71.9%), P = 0.027. The incidence of other adverse events and infections after PSE was not statistically different.

**Conclusion:**

PSE with 8Spheres is safe and effective. The use of 500–700 um microsphere for PSE can make the increase of peripheral blood cells more stable. Each vial of 8Spheres corresponds to a certain volume of splenic embolization, so it is possible to achieve quantitative embolization in PSE.

## Introduction

Hypersplenism is a clinical syndrome that is mainly characterized by splenomegaly and cytopenias. Hypersplenism is mostly associated with splenomegaly. Splenomegaly can be divided into primary and secondary. Primary splenomegaly is rare in clinical practice, and the cause of the disease is not yet clear. The causes of secondary splenomegaly are cirrhosis, portal hypertension, infectious diseases, immune diseases, hematological diseases, and splenic proliferative diseases. In China, cirrhosis is the most important cause of hypersplenism. Patients with hypersplenism have cytopenias, anemia, decreased immune function, infection, bleeding, and so on. The main treatments for hypersplenism include medical treatment, surgical treatment and interventional therapy [1]. Among the interventions, partial splenic embolization (PSE) was first reported in 1973 [2] and is accepted by more and more physicians and patients due to its minimal invasiveness and rapid recovery [3–6]. Partial splenic embolization (PSE), not only increases peripheral blood cells, but also effectively reduces portal venous pressure and treats gastroesophageal variceal bleeding due to cirrhosis [7, 8]. Many studies have reported that the efficacy and complications of partial splenic embolization are related to the volume of splenic embolization. The larger the volume of splenic embolization, the better its efficacy, but the higher the incidence of complications. The smaller the volume of splenic embolization, the lower the incidence of postoperative complications, but the efficacy is poor. It is very difficult to accurately grasp the embolization volume during partial splenic embolization, so the risk of postoperative complications is high, and partial splenic embolization cannot be standardized. At present, some commonly used embolization materials for splenic embolization include microspheres, gelatin sponge particles, PVA particles and coils. 8Spheres is a new type of polyvinyl alcohol microspheres, which is composed of polyvinyl alcohol backbone, covalent bond form and cross-linking agent. It has different particle size models and is loaded with 1 g microspheres in each bottle. The aim of this study was to investigate the safety and efficacy of 8Spheres in partial splenic embolization. Since the number of microspheres contained in each bottle of 8 spheres was fixed and moderate, it was explored whether the possibility of accurate control of splenic embolic volume could be achieved by quantifying the number of microspheres used during PSE.

## Materials and methods

### General information

The data of 179 patients who underwent partial splenic embolization in the Department of Radiology, Union Hospital, Tongji Medical College, Huazhong University of Science and Technology from January 2017 to January 2020 were collected. Inclusion criteria (1) clinical and imaging diagnosis of liver cirrhosis, hypersplenism; (2) liver function Child–Pugh class A or B, performance score (ECOG) 0–2 points; (3) blood routine: leucocytes ≤ 3.0 × 10^9^/L, platelets ≤ 50 × 10^12^/L; (4) aged 18–70 years old; (5) no history of chemoradiotherapy, immunotherapy, molecular targeted drugs, etc. Exclusion criteria: (1) Child–Pugh class C liver function, performance score (ECOG) ≥ 3 points; (2) severe coagulation dysfunction and can not be corrected; (3) accompanied by gastrointestinal bleeding or malignant tumors; (4) a large number of ascites; (5) combined with infection; (5) severe heart, brain, lung, renal insufficiency. The patients were divided into two groups according to the size of microspheres used in PSE: 300–500 um microsphere group (N = 83) and 500–700 um microsphere group (N = 96). The baseline data before PSE included: gender, age, etiology of liver cirrhosis, preoperative Child–Pugh classification of liver function, ECOG score, ALT, AST, total bilirubin, leucocyte count, platelet count, spleen volume, etc.

### Method

After disinfection, draping, and local anesthesia of the puncture site with 2% lidocaine, the right femoral artery was punctured using the Seldinger technique and a 5F vascular sheath was placed. 5F Yashino catheter was used to cannulate the celiac trunk and splenic artery for angiography. After the course of splenic artery was confirmed, the catheter was cannulated to the end of splenic artery trunk. Angiography was repeated to confirm the absence of vascular branches other than the spleen, such as vessels of the stomach, pancreas, omentum, etc. If there are arterial branches outside the spleen, the catheter tip must extend beyond the opening of these arteries. One bottle of 8Spheres microspheres (containing 1 g microspheres) + contrast agent suspension was slowly injected for embolization. Use a 20 ml syringe to prepare the microspheres and contrast medium into suspension in the proportion of 1:1. Use a 2 ml syringe to connect a 20 ml syringe through a three-way connection, and suck 2 ml microspheres at each time. Slowly inject under fluoroscopy at an injection rate of 2 ml/min to prevent reflux. The angiography was reexamined to assess the splenic embolization volume. The sizes of microspheres used in PSE are 300-500 um and 500-700 um. The incidence of adverse events was observed after operation, and the evaluation standard was Common Terminology Criteria for Adverse Events (CTCAE4.0). The pain of patients within 10 days after operation was evaluated by VAS visual analogue scale. The volume software of Siemens CT workstation was used to measure the spleen volume of patients. After manual labeling layer by layer, the software automatically calculated the measured volume. The volume of spleen before operation, the volume of spleen rechecked one month after operation and the volume of infarcted area of spleen were measured.

The patients were reexamined at 1, 3, 6, 9 and 12 months after operation. Blood routine and other indicators were detected.

Materials used for PSE: 5F vascular sheath (TERUMO5F-10CM, Terumo, Japan), 0.035 inch (RFGA35153M, Terumo, Japan), 5F Yashino catheter (Terumo, Japan), 2.7F microcatheter (Terumo, Japan), 8Spheres (Suzhou Hengrui callisyn Biomedical Technology Co., Ltd, China).

### Outcome measures

1. The volume of spleen, the volume of splenic infarct area and the rate of splenic infarction were reexamined 1 month after operation in both groups.
2. The incidence of adverse events after PSE in the two groups; (including abdominal pain, fever, vomiting, pleural effusion, ascites)
3. The changes of leucocyte and platelet during postoperative follow-up in the two groups;
4. Abdominal pain within 10 days after operation in both groups;
5. The incidence of postoperative infection in the two groups.

### Statistical methods

Statistical analysis was performed using SPSS software (Version 24.0, IBM, Armonk, New York). Number of cases (expressed as percentage) was used for enumeration data, and chi-square test was used for differences, including Pearson Chi-Square and Fisher's Exact Test. Measurement data were expressed as mean ± standard deviation, and two independent samples t-test was used. P < 0.05 was considered to indicate a statistically significant difference.

## Results

### Basic information

#### Comparison of preoperative enumeration data between the two groups

There was no statistically significant difference in gender, Child–Pugh classification of liver function, or etiology of cirrhosis between the two groups.

As shown in Table 1, Chi-square test was used between two groups with P value > 0.05 and no statistical difference.

**Table 1:** General information of the patients

		Group		Chi-square test (p-value)	
		300–500 um group (N = 83)	500–700 um group (N = 96)	Pearson Chi-square	Fisher's exact test
Gender					
Male	Count (%)	51 (61.4%)	61 (63.5%)	0.295	0.376
Female	Count (%)	32 (38.6%)	35 (36.5%)		
Child–Pugh classification of liver function					
A	Count (%)	63 (75.9%)	74 (77.1%)	0.349	0.416
B	Count (%)	20 (24.1%)	22 (22.9%)		
Etiology of cirrhosis					
Hepatitis B	Count (%)	59 (71.1%)	67 (69.8%)	0.447	
Hepatitis C	Count (%)	13 (15.7%)	16 (16.7%)		
Alcoholic cirrhosis	Count (%)	8 (9.6%)	10 (10.4%)		
Autoimmune cirrhosis	Count (%)	3 (3.6%)	3 (3.1%)		
ECOG score					
0	Count (%)	48 (57.8%)	51 (53.1%)	0.305	
1	Count (%)	24 (28.9%)	29 (30.2%)		
2	Count (%)	11 (13.3%)	16 (16.7%)		

#### Comparison of preoperative measurement data between the two groups

There was no statistically significant difference in age, ALT, AST, Bilirubin, leukocyte count, platelet count, and volume of the spleen between the two groups before PSE.

As shown in Table 2, t test was used for comparison between two groups, with P value > 0.05, without statistical difference.

**Table 2:** Patient's age, liver function, blood routine and Spleen Volume before PSE

Group	Mean	SD	Levene's test for equality of variances (p-value)	t-test (p-value)
Age				
300–500 um group	48.1	13.6	0.145	0.329
500–700 um group	47.4	14.2		
Preoperative ALT				
300–500 um group	32.7	18.1	0.357	0.462
500–700 um group	31.8	19.3		
Preoperative AST				
300–500 um group	29.4	19.9	0.271	0.344
500–700 um group	33.6	16.9		
Preoperative Bilirubin				
300–500 um group	16.9	9.8	0.134	0.228
500–700 um group	18.1	8.3		
Preoperative leukocyte count				
300–500 um group	1.98	0.49	0.437	0.592
500–700 um group	2.03	0.37		
Preoperative platelet count				
300–500 um group	35.6	9.8	0.486	0.664
500–700 um group	34.1	10.2		
Preoperative Spleen Volume (cm^3^)				
300–500 um group	1279.36	378.62	0.263	0.351
500–700 um group	1198.71	415.37		

### Comparison of preoperative spleen volume, postoperative spleen volume, spleen embolism volume and spleen embolism rate between the two groups

The volume of the spleen before PSE was not statistically different between the two groups. There was a statistically significant difference in spleen volume after PSE between the two groups, which was less in the 500–700 um group than in the 300–500 um group. There was a statistically significant difference in the volume of splenic infarction after PSE between the two groups, which was greater in the 500–700 um group than in the 300–500 um group.

As shown in Table 3, comparison between two groups using t-test, P value < 0.05, statistically significant difference.

**Table 3:** Comparison of preoperative spleen volume, postoperative spleen volume, spleen embolization volume and spleen embolization rate between the two groups

Group	Mean	SD	Levene's test for equality of variances (p-value)	t-test (p-value)
Preoperative spleen volume				
300–500 um group	1279.36	378.62	0.263	0.351
500–700 um group	1198.71	415.37		
Postoperative spleen volume				
300–500 um group	753.82	325.41	0.008	0.011
500–700 um group	568.65	298.16		
Spleen embolization volume				
300–500 um group	525.93	118.29	0.014	0.025
500–700 um group	630.26	109.71		
Spleen embolization rate				
300–500 um group	41.1	12.3	0.021	0.030
500–700 um group	52.4	10.1		

### Changes of leukocytes and platelets at different stages after operation in the two groups

(1) leukocytes

In the first week the increase in leukocytes was more significant in the 300–500 um group than in the 500–700 um group, with a statistically significant difference. In the first and third months, the leukocytes elevation in the 500–700 um group was more significant than that in the 300–500 um group, with a statistically significant difference. There was no statistical difference between the two groups at 6–12 months, but the absolute value was higher in the 500–700 um group than in the 300–500 um group. The leukocytes in the 300–500 um group showed a "rapid rise and rapid fall" type; the leukocytes in the 500–700 um group showed a "rapid rise and slow fall" type.

(2) platelets

There was no statistically significant difference in platelet elevation between the 300–500 um group and the 500–700 um group in the first week. In the first and third months, the platelet increase in the 500–700 um group was more significant than that in the 300–500 um group, with a statistically significant difference. There was no statistical difference between the two groups at 6–12 months. Platelets in the 300–500 um group showed a "fast rise and fast fall" type; platelets in the 500–700 um group showed a "fast rise and slow fall" type.

As shown in Table 4, comparison between two groups using t-test, P value < 0.05, statistically significant difference.

**Table 4:** Comparison of leukocytes and platelets at different stages after PSE between the two groups

	Group		Chi-square test (p-value)	
	300–500 um group (N = 83)	500–700 um group (N = 96)	Pearson Chi-square	Fisher's exact test
1 week				
Postoperative leukocyte count	5.33 ± 1.26	5.26 ± 1.32	0.321	0.397
Postoperative platelet count	98.3 ± 13.2	101.2 ± 16.1	0.463	0.517
1 month				
Postoperative leukocyte count	4.13 ± 0.91	5.08 ± 1.16	0.026	0.031
Postoperative platelet count	125.6 ± 20.3	138.7 ± 18.4	0.019	0.025
3 months				
Postoperative leukocyte count	4.08 ± 1.25	4.83 ± 0.98	0.022	0.036
Postoperative platelet count	121.8 ± 16.9	134.3 ± 20.1	0.017	0.023
6 months				
Postoperative leukocyte count	3.86 ± 0.89	4.01 ± 0.67	0.098	0.105
Postoperative platelet count	110.6 ± 15.3	115.7 ± 14.9	0.086	0.093
9 months				
Postoperative leukocyte count	3.73 ± 0.85	3.82 ± 0.72	0.257	0.342
Postoperative platelet count	101.1 ± 21.6	102.4 ± 18.8	0.353	0.448
12 months				
Postoperative leukocyte count	3.61 ± 0.59	3.65 ± 0.70	0.583	0.679
Postoperative platelet count	90.4 ± 19.3	89.7 ± 21.2	0.614	0.703

### Comparison of postoperative adverse reactions and infection between the two groups

The incidence of abdominal pain after PSE was statistically different between the two groups, it was higher in the 300–500 um group than in the 500–700 um group. There was no statistical difference in fever, vomiting, hydrothorax, and Ascites. The incidence of infection after PSE was also not statistically different between the two groups.

As shown in Table 5, Chi-square test was used for comparison between two groups, including Pearson Chi-Square and Fisher's Exact Test, and P value < 0.05 was statistically significant.

**Table 5:** Incidence of adverse events and Infection after PSE

		Group		Chi-square test (p-value)	
		300–500 um group (N = 83)	500–700 um group (N = 96)	Pearson Chi-square	Fisher's exact test
Postoperative fever					
No	Count (%)	14 (16.9%)	19 (19.8%)	0.502	0.613
Yes	Count (%)	69 (83.1%)	77 (80.2%)		
Postoperative vomiting					
No	Count (%)	58 (69.9%)	71 (74.0%)	0.224	0.351
Yes	Count (%)	25 (30.1%)	25 (26.0%)		
Postoperative abdominal pain					
No	Count (%)	11 (13.3%)	27 (28.1%)	0.027	0.032
Yes	Count (%)	72 (86.7%)	69 (71.9%)		
Postoperative Infection					
No	Count (%)	83 (100%)	95 (99.0%)	0.746	0.875
Yes	Count (%)	0 (0%)	1 (1.0%)		
Hydrothorax					
No	Count (%)	66 (79.5%)	74 (77.1%)	0.496	0.535
Yes	Count (%)	17 (20.5%)	22 (22.9%)		
Ascites					
No	Count (%)	60 (72.3%)	68 (70.8%)	0.557	0.682
Yes	Count (%)	23 (27.7%)	28 (29.2%)		

### Comparison of VAS score of abdominal pain within 10 days after operation between the two groups

There was a statistically significant difference in VAS score for abdominal pain between the two groups on days 3–5 after PSE, it was higher in the 300–500 um group than in the 500–700 um group. There was no statistical difference in VAS score of abdominal pain between the two groups at other time points.

As shown in Table 6, comparison between two groups using t-test, P value < 0.05, statistically significant difference.

**Table 6:** VAS score of postoperative abdominal pain in both groups

	Group		Chi-square test (p-value)	
	300–500 um group (N = 83)	500–700 um group (N = 96)	Pearson Chi-square	Fisher's exact test
Day 1	1.8 ± 1.3	1.6 ± 1.4	0.326	0.439
Day 2	2.1 ± 1.2	1.9 ± 1.1	0.215	0.331
Day 3	8.6 ± 1.0	7.3 ± 1.2	0.029	0.031
Day 4	7.8 ± 1.1	6.1 ± 1.0	0.007	0.009
Day 5	5.5 ± 0.9	4.2 ± 1.1	0.016	0.021
Day 6	3.3 ± 1.3	3.1 ± 1.2	0.347	0.416
Day 7	2.4 ± 1.0	2.3 ± 0.8	0.442	0.503
Day 8	2.1 ± 0.8	2.0 ± 0.7	0.513	0.538
Day 9	1.9 ± 1.2	2.0 ± 1.1	0.694	0.732
Day 10	1.7 ± 1.3	1.8 ± 1.2	0.681	0.711

## Discussion

Hypersplenism is a clinical syndrome dominated by decreased blood cells due to a variety of causes, hypersplenism is mostly associated with splenomegaly. Splenomegaly can be divided into primary and secondary causes. Its pathogenesis is due to the significantly enhanced phagocytosis of monocyte-macrophages accompanied by the destruction of blood cells after spleen enlargement, while a large number of blood cells are retained in the hyperplastic splenic sinus, which causes peripheral blood cytopenias [9–11]. The treatment of hypersplenism includes medical treatment, surgical treatment, and interventional therapy [12]. Medical treatment is mainly the use of drugs that stimulate blood cell growth, with good short-term efficacy and poor long-term efficacy. Surgical treatment is mainly splenectomy, which has a greater impact on the patient's immune function and may lead to fulminant infection [13], and splenectomy may lead to portal vein thrombosis [14], resulting in or aggravating gastrointestinal bleeding in patients. Interventional therapy includes partial splenic embolization, splenic ablation [15], and so on. Splenic embolization was first introduced in 1973, when autologous blood clot was used by Maddison [16] to produce splenic artery embolization for hypersplenism treatment. Seven years later, transcatheter partial splenic embolization (PSE) was developed by Spigos et al. [17], which has been proved as a safe and effective method of vascular occlusion. Partial splenic embolization is usually performed by inserting a catheter through the femoral artery or radial artery approach [18]. The injection of embolic agent through the catheter causes necrosis of part of the splenic tissue, reduces the volume of the spleen, reduces the retention of blood cells, reduces the phagocytic and destructive ability of monocyte-macrophages, and thus increases the number of peripheral blood cells. Splenic ablation is performed by cryoablation or microwave ablation, which directly leads to local necrosis of the spleen and achieves the effect of reducing the size of the spleen, but the risk is high. Shoufei Jiao et al.[19] reported that PSE is simple and minimally invasive and is better than splenectomy. Therefore, for the interventional treatment of hypersplenism, partial splenic embolization is still the main treatment. Partial splenic embolization can effectively increase peripheral blood cells and reduce portal venous pressure under the condition of preserving splenic function. Jiangtao Liu et al. [20] found that PSE decreases both the wedged hepatic venous pressure and the hepatic venous pressure gradient (HVPG). Yiming Zhao et al. [21] reported that PSE immediately reduced the portal pressure, and HVPG remained stable at 6 months after surgery. PSE is considered as a safe and easy to implement method, and is expected to be one of the treatments for reducing the portal pressure. Jennifer Vittorio [22] found that PSE is a safe and effective alternative in the management of pediatric portal hypertension in children.

Studies have shown that the volume of splenic embolization is associated with efficacy, side effects, and complications. The smaller the volume of splenic embolization, the milder the postoperative side effects and the lower the incidence of complications, but the efficacy is poor. The larger the volume of splenic embolization, the more obvious the curative effect, but the side effects are serious, and the incidence of complications is significantly increased. Abhinav Talwar et al. [23] reported that underlying liver dysfunction and high infarction rates may be risk factors leading to major complications. They suggest that interventional radiologists should be aware of the complication profile of this procedure and further advance research in techniques dealing with hypersplenism. Tyson A Hadduck et al. [24] found that the rate of complications has been shown to increase as the percent of total splenic volume embolized increases. Studies have shown that splenic embolization volume is an important factor affecting efficacy, and it is generally recommended to control splenic embolization volume at 40–70% [25–27]. Takashi Tajiri et al. [28] reported that hypersplenism can be relieved effectively by this material when the embolized area of spleen exceeds 50%. Although partial splenic embolization is technically very mature, the difficulty in practical operation is to accurately grasp the embolization volume during PSE, find a balance point in the efficacy and side effects, and reduce the incidence of complications. At present, the commonly used evaluation method is intraoperative reexamination of angiography, and the embolization volume is inferred by the display of splenic artery branches after embolization [29]. The embolization volume judged by this method is often different from the embolization volume evaluated according to the image data reexamined after operation. In recent years, with the development of interventional equipment and software, many scholars are also exploring new and more accurate evaluation methods. Toru Ishikawa et al. [30] found that the splenic embolization ratio measurement obtained via cone beam CTA can be used to assess PSE treatment endpoints. Ming-Ching Ou et al. [31] reported that Estimated splenic embolization volume was calculated by a method based on diameters of the splenic artery and its branches which were measured via 2D angiographic images, the method provides a simple method to quantitatively estimate embolized splenic volume. Jun Koizumi et al. [32] found that the use of diffusion-weighted imaging (DWI) for estimating infarcted splenic volume during partial splenic embolisation (PSE) allowed semi-automated splenic volumetry on site. It was reported by Akihiko Osaki [33] that platelet count after PSE can be predicted before the procedure by using the Vh (The hepatic volume)/Vsp (splenic volume) ratio and the anticipated spleen embolization volume, which can prevent too much or too little embolization, thereby leading to an improvement in the risk/return trade-off in PSE. Different literatures on the selection of embolization materials for partial splenic embolization have also been reported differently [34, 35], in addition to the commonly used PVA particles, gelatin sponge particles, and microspheres, there are reports on the use of coils for PSE [36]. 8Spheres is a new type of polyvinyl alcohol embolic microspheres, which have different particle size models and are loaded with 1 g microspheres per vial. Because of its regular and spherical shape, and its variable and compressible shape, it is not easy to block the tube and can walk further in the vessel. This study found that there was no significant difference in the volume of spleen before PSE between the two groups, but there was significant difference in the volume of spleen after PSE. The volume of spleen after PSE in 500–700 um group was smaller. There was also significant difference in the volume of splenic infarction after PSE between the two groups. The infarct volume was larger in the 500-700 um group. A bottle of microspheres was used in both groups of patients, with a microsphere content of 1 g. The embolization volume and embolization rate were higher in the 500–700 um group. It may be tha larger particle size microspheres can embolize larger diameter vessels, resulting in larger volume of splenic infarction, but both 300–500 um and 500–700 um particle size microspheres can well cause splenic infarction. Since the content of 8Spheres in each vial is fixed, moderate and well prepared into a mixture, each vial of different sizes of embolic microspheres has the corresponding splenic embolization volume. If the volume of spleen before embolization is measured by CT before operation, the volume of spleen that needs embolization is calculated. It is possible to calculate the amount of microspheres to be used and to achieve quantitative embolization of the spleen. The theoretical basis of PSE is derived from the anatomy of the spleen [37], which is composed of white pulp, red pulp, and marginal zone. The red pulp is located peripherally and is composed of splenic cords and sinusoids. The red pulp has a large number of phagocytes and is also an important location for storing blood. In hypersplenism, a large number of blood cells stasis in the red pulp, resulting in peripheral blood cytopenias, such as leukocytes, platelets. Partial splenic embolization is the use of particles with appropriate size to embolize distal branches of the central artery, reduce the volume of red pulp, and achieve the effect of increasing peripheral blood cells. Gisèle N'Kontchou et al. [38] reported that PSE may resolve cytopenia and the clinical complications related to hypersplenism or splenomegaly in patients with cirrhosis. Birger Pålsson et al. [39] found that Partial splenic embolization (PSE) by the injection of microspheres via a catheter comprising approximately 30–70% of the splenic parenchyma is now a safe method, which significantly reduces the cytopenia induced by hypersplenism, especially thrombocytopenia. Therefore, partial splenic embolization can not only effectively increase peripheral blood cells, but also preserve the function of the spleen and activate the immune function of the body. Yasushi Matsukiyo et al. [40] reported that PSE not only promoted the recovery of leukopenia and thrombocytopenia but also induced activation of host immunity in patients with cirrhosis and thrombocytopenia. Maria Passhak et al. [41] found that PSE can be considered as a treatment option for hypersplenism-related thrombocytopenia. It was reported by Secil Omer [42] that PSE provided a safe alternative to surgery for treating thrombocytopenia in patients with liver cirrhosis. Partial splenic embolization is effective not only for hypersplenism due to cirrhosis but also for thrombocytopenia due to ITP. Emi Togasaki et al. [43] found that PSE is a safe and effective alternative therapy to splenectomy for patients with steroid-resistant ITP as it generates long-term, durable responses.

In this study, we found that after partial splenic embolization with 8Spheres, the leukocytes and platelets in 300-500 um group and 500-700 um group were significantly increased from 1 week to 1 year after operation. There was a statistically significant difference between the two groups. The number of leukocytes and platelets in 500-700 um group was higher than that in 300-500 um group. At other follow-up times, leukocytes and platelets were not statistically different between the two groups. The reason may be that although the number of microspheres used is the same, embolization with a particle size of 500–700 um can cause a higher volume of splenic infarction, a smaller volume of non-infarcted spleen, and a greater reduction in the volume of splenic red pulp. Mingyue Cai et al. [44] found that Linear mixed model analysis indicated the splenic infarction ratio (P < 0.001), non-infarcted splenic volume (P = 0.012), and cholinesterase level (P < 0.001) were significantly associated with the platelet increment after PSE. Masaki Nio et al. [45] reported that a rise in platelet count would be found 12 to 24 h after PSE, while the peak count (beyond the lower limit of normal level) will be reached in 1 or 2 weeks. This is generally consistent with our findings. It was reported by Takashi Tajiri [28] that platelet counts increased maximally by 2 weeks after embolization, followed by a gradual decrease, platelets remained significantly more numerous than before embolization for up to 8 years. The results of our study showed that the platelet count of patients increased to the peak in one month, and then decreased gradually. However, after one year of follow-up, the platelet count of patients was still significantly higher than that before operation, which was similar to the results of Takashi Tajiri study. There are many studies on the choice of embolic material and particle size in partial splenic embolization [46–49]. The microsphere with the size of 300-500 um can reach the distal end of the blood vessel and achieve the terminal embolization, which is not easy to form collateral circulation and reduce the recurrence of hypersplenism. The common side effects and complications of partial splenic embolization include post embolization syndrome (such as abdominal pain, fever, vomiting), portal vein thrombosis, liver failure, pleural effusion, ascites and splenic abscess [38, 50–53]. Gabriele Piffaretti et al. [54] reported that postembolization syndrome is common and has been reported as 30% but generally resolving without sequelae. This study showed that the incidence of postoperative abdominal pain in the two groups with statistical difference; There was no significant difference in the incidence of other side effects and infection between the two groups. R Shah et al. [55] found that abdominal pain and fever have been reported as 82% and 94%. The incidence of side effects and infection after splenic embolization in this study is basically consistent with other related studies. The incidence of side effects and infection after splenic embolization is positively correlated with splenic embolization volume, so it is very important to control splenic embolization volume accurately. M Obatake et al. [56] suggested that the first embolization volume of the spleen should not exceed 70% of the spleen volume, so as to reduce the incidence of complications. After splenic embolization in both groups, the most frequent side effect was abdominal pain. In the VAS visual analogue scale of abdominal pain at 1–10 days after operation in the two groups, the VAS score at 3–5 days after operation in the 300–500 um group was higher than that in the 500–700 um group, with statistically significant difference. The reason may be that different particle sizes of 8Spheres reach different levels of embolization. 300-500 um microspheres can reach the branches below the central arteriole of the spleen and near the distal red pulp. There is no collateral circulation here, which realizes the complete embolization of the splenic vessels. The aseptic inflammatory reaction caused by the necrotic substances is large, so the degree of postoperative abdominal pain is serious. Chao et al. [57] showed that the embolic material was the main influencing factor of post embolism syndrome. The smaller the embolic particle size, the earlier and more severe the abdominal pain.

## Conclusions

PSE using 8Spheres microspheres at the end of the splenic artery trunk is safe and effective. Compared with the use of 300-500 um microsphere, the use of 500-700 um microsphere for partial splenic embolization can make the increase of peripheral blood cells more stable. Each bottle of 8Spheres microspheres contained a fixed number of microspheres, and each bottle of microspheres corresponded to a certain volume of splenic embolization. The volume of spleen can be measured by preoperative CT, the volume of spleen planned for embolization can be calculated. The amount of microspheres needed in the procedure can be calculated based on the volume of spleen planned for embolization. Therefore, it is promising to achieve the purpose of quantitative embolization in PSE. The shortcoming is that this is a retrospective study and the data is from a single center. A multicenter, large-sample, prospective study can be conducted in the future, and a controlled study can be designed to compare with the embolization materials commonly used in PSE, so as to provide more help for clinical work.

## Data Availability

The datasets used and analysed during the current study are available from the corresponding author on reasonable request.
